# Genetic Ablation of *Ankrd1* Mitigates Cardiac Damage during Experimental Autoimmune Myocarditis in Mice

**DOI:** 10.3390/biom12121898

**Published:** 2022-12-18

**Authors:** Ieva Rinkūnaitė, Egidijus Šimoliūnas, Milda Alksnė, Gabrielė Bartkutė, Siegfried Labeit, Virginija Bukelskienė, Julius Bogomolovas

**Affiliations:** 1 Department of Biological Models, Institute of Biochemistry, Life Sciences Center, Vilnius University, LT-10257 Vilnius, Lithuania; 2 DZHK Partner Site Mannheim-Heidelberg, Medical Faculty Mannheim, University of Heidelberg, 68167 Mannheim, Germany; 3 Myomedix GmbH, 69151 Neckargemünd, Germany; 4 Department of Medicine, School of Medicine, University of California, San Diego (UCSD), La Jolla, CA 92093, USA

**Keywords:** myocarditis, dilated cardiomyopathy, ANKRD1, myocardial remodeling, inflammation, heart failure

## Abstract

Myocarditis (MC) is an inflammatory disease of the myocardium that can cause sudden death in the acute phase, and dilated cardiomyopathy (DCM) with chronic heart failure as its major long-term outcome. However, the molecular mechanisms beyond the acute MC phase remain poorly understood. The ankyrin repeat domain 1 (ANKRD1) is a functionally pleiotropic stress/stretch-inducible protein, which can modulate cardiac stress response during various forms of pathological stimuli; however, its involvement in post-MC cardiac remodeling leading to DCM is not known. To address this, we induced experimental autoimmune myocarditis (EAM) in ANKRD1-deficient mice, and evaluated post-MC consequences at the DCM stage mice hearts. We demonstrated that ANKRD1 does not significantly modulate heart failure; nevertheless, the genetic ablation of *Ankrd1* blunted the cardiac damage/remodeling and preserved heart function during post-MC DCM.

## 1. Introduction

Myocarditis (MC) is an inflammatory disease of the myocardium with an incidence of 3,071,000 cases per year worldwide, which significantly contributes to the global burden of cardiovascular diseases [1]. MC typically presents as a rapid-onset severe hemodynamic instability often leading to sudden death in the acute phase, and subsequently resulting in dilated cardiomyopathy (DCM) with chronic heart failure (HF) as the major long-term sequelae [2,3,4]. MC can be caused by a variety of infectious (e.g., viral, bacterial) and noninfectious agents (including cardiotoxic drugs, hypersensitivity reactions, systemic disorders, and radiation) [5,6,7].

The interplay between the activated immune system and cardiac response to inflammation is at the core of MC pathophysiology [6,8,9]. Cardiac inflammation activates complex signaling pathways that generate numerous compensatory changes affecting the geometry, size, and function of the heart, which often become maladaptive [6,7,8,9]. Understanding these signaling pathways would open new therapeutic, prognostic, and diagnostic prospects for MC and its outcome.

Ankyrin repeat domain 1 (ANKRD1, also known as cardiac ankyrin repeat protein, CARP) is a highly conserved member of the muscle ankyrin repeat protein (MARPs) family of stress-inducible proteins [10]. ANKRD1 is predominantly expressed in adult cardiomyocytes and normally is located at the I-band of the sarcomere as a component of a titin-associated stretch-sensing complex [11,12]. *ANKRD1* transcript and protein levels in the postnatal heart rapidly react to various hemodynamic insults, stretch, and cardiomyopathies of different etiologies [13,14,15,16,17], acting as a part of program that is induced during cardiac remodeling [14,18]. Global murine *Ankrd1* knockouts are viable and exhibit no basal phenotype [19,20]. However, genetic ablation of *Ankrd1* prevents DCM phenotype development in a genetic model of DCM [21] and attenuates chemically induced cardiac hypertrophy [22]. Thus, we hypothesized that ANKRD1 could also modulate development and progression of MC.

To address the role of ANKRD1 in the pathophysiology of MC-induced DCM, we used a well-established experimental autoimmune myocarditis (EAM) model mimicking human fulminant MC in the acute phase and human DCM in the late phase [23,24], and evaluated the cardiac stress response in ANKRD1-deficient mice at DCM stage. In this work, we demonstrated that ANKRD1 is not an essential modulator of HF; nevertheless, genetic ablation of *Ankrd1* had a beneficial effect on post-MC cardiac remodeling.

## 2. Materials and Methods

### 2.1. Experimental Animals

Genetically modified knock-out mice with disruption of their *Ankrd1* gene (*Ankrd1*^tm1(KOMP)Vlcg^; MGI:3808333) were generated at the University of Arizona BIO5 Genetically Engineered Mouse Models (GEMM) Core from embryonic stem cells (10563B-B6 (VGB6); strain of origin C57BL/6NTac) obtained from the KOMP consortium (Bar Harbor, ME, USA). Briefly, the *Ankrd1* gene (encoding the ANKRD1 protein) was targeted using the VelociGene technique with the ZEN-UB1 cassette (which disrupts the *Ankrd1* gene while simultaneously inserting a *lacZ* gene, which should serve as an enhancer trap). The generated mouse strain was backcrossed onto BALB/c genetic background (due to C57BL/6 resistance to experimental autoimmune myocarditis (EAM) [25]) (at University of Heidelberg, Medical Faculty Mannheim, Germany; Vet. Approval No 35-9185.64/BH ZMF) via a speed congenic approach (Taconic Biosciences, Rensselaer, NY, USA). Further *Ankrd1^−/−^* mice strains were maintained by brother/sister mating and constant genetic control at the Vilnius University, Institute of Biochemistry, Lithuania (Vet. Approval No LT 59–13-001, LT 60–13-001, LT 61–13-004). Validation of ANKRD1 loss in *Ankrd1^−/−^* mice is provided in Figure S1, Supplementary Materials. Primers used in PCR for mice colony genotyping (Table S1, Supplementary Materials) were purchased from Metabion International AG (Planegg/Steinkirchen, Germany). Experiments were performed on same generation wild-type (*n* = 13) and knock-out (*n* = 16) 7- to 8-week-old littermate male mice. Experimental groups were formed using the resource equation method. Animals were supervised daily and maintained under standard controlled conditions: temperature 22  ±  1  °C, humidity 55  ±  3%, and a 12  h light/12  h dark cycle. They were fed with standard commercial rodent feed (Altromin, Spezialfutter GmbH & Co. KG, Lage, Germany) and had access to water *ad libitum*. No animals died before the experiment endpoint and no animals were excluded from experiments or data analysis. Animals were euthanized with a flow of 8.0  L/min of medical CO_2_ gas (Elme Messer Lit, Vilnius, Lithuania) followed by cervical dislocation. All experimental procedures conformed to Directive 2010/63/EU requirements and were approved by the Lithuanian State Food and Veterinary Service (Approval No G2-176, 22/03/2021) and the Ministry of Environment of the Republic of Lithuania (Notification No RN-33, 08/04/2020).

### 2.2. Immunization

The induction of EAM was performed as described [23,26,27]. Briefly, an α-myosin heavy chain (α-MHC) α614–629 (NH_2_-SLKLMATLFSTYASAD-COOH) peptide derived from murine hearts (INTAVIS Bioanalytical Instruments AG, Cologne, Germany) was used for immunization. Cardiac myosin serves as an autoantigen and provokes autoimmunity, which leads to the subsequent progression of acute MC into DCM. The peptide was dissolved at 1 mg/mL in sterile phosphate-buffered saline (PBS) (Gibco, Carlsbad, CA, USA) and emulsified on ice 1:1 (*v*/*v*) with Freund’s complete adjuvant (CFA) (supplemented with 5 mg/mL of *Mycobacterium tuberculosis*) (Chondrex, Inc., Abingdon, UK). On day 0, anesthetized (randomly grouped) WT (*n* = 8) and *Ankrd1* KO (*n* = 11) mice were immunized s.c. with 150 μg/animal peptide emulsified in CFA. On the same day, all mice were additionally injected i.p. with 500 ng of *pertussis toxin* (Invitrogen, Carlsbad, CA, USA) prepared in 0.9% sterile saline (B.Braun Medical, Melsungen, Germany) and supplemented with 1% bovine serum albumin (BSA) (Gibco, Carlsbad, CA, USA). On day 7, mice were boosted by s.c. injection with 150 μg/animal peptide emulsified in CFA (5 mg/mL of *Mycobacterium tuberculosis*). Sham animals—WT (*n* = 5) and *Ankrd1* KO (*n* = 5)—were immunized following the same protocol described above but instead of the peptide, PBS mixed 1:1 (*v*/*v*) with CFA was used for both immunizations. 

### 2.3. Echocardiography

The procedure was performed 0, 21, 40, and 65 days post-immunization (pi). The selected timeframe for the experiment encloses the well-established myosin-induced acute MC (21 days pi) followed by DCM in the late phase (40–70 days pi) model. Animals were lightly anesthetized with 1% isoflurane (Vetpharma AH, Barcelona, Spain) (in pure oxygen (Elme Messer Lit, Vilnius, Lithuania) 0.6 L/min) using the EZ-SA800 anesthesia system (E-Z Systems, Palmer, PA, USA) and positioned on a pre-heated table (37 °C) after skin preparation. Transthoracic two-dimensional (2D) guided M-mode echocardiography on the mouse was performed using a HITACHI EUB-7000HV (Hitachi, Ltd., Tokyo, Japan) ultrasound system with a 13 MHz linear probe. Heart rates were kept consistent between experimental groups (500–700 bpm). The heart was imaged with the 2D mode at the parasternal short-axis view at the level of the papillary muscles. The M-mode cursor line was positioned through the left ventricle (LV) largest dimension for the assessment of LV chamber dimensions and systolic function. Image depth, width, and gain setting adjustments were used to optimize image quality; all mice were tested using the same parameters. LV end-diastolic dimension (LVEdD) and LV end-systolic chamber dimension (LVEsD) were measured from B-mode recordings. Five cardiac cycles per measurement were used for averaging. The percentage of LV fractional shortening (FS) was calculated as [(LVdD - LVsD)/LVdD] X 100 and was used as an indicator of systolic cardiac function. Data analysis was performed using the HITACHI EUB-7000HV instrument by an operator blinded to the experimental group.

### 2.4. Body, Heart, and Lung Weights

During the experiment (before immunization and until 65 days pi) mice were weighed twice a week (Figure S2, Supplementary Materials). The 65 days pi (at the DCM phase) mice were sacrificed, then the hearts and lungs were rapidly dissected, placed on ice, and rinsed in ice-cold PBS. After, they were drained by gently squeezing on absorbent paper. Organs were weighed and the heart and lung weights (mg) were normalized by the tibia bone length (TL) (mm).

### 2.5. Histology

After dissection, hearts were blindly scored by two independent researchers. Damage severity grading (for the gross score) was evaluated as described [23] by the following scoring system: 0—no visible damage of the heart; 1—less than 10%; 2—10–30%; 3—30–50%; 4—50–90%; 5—more than 90%. At first, the atria were removed and then hearts were cut transversely at the mid-ventricular level. One portion of the heart was snap-frozen in liquid nitrogen and stored at −80 °C for further analysis. Another portion of the heart, and part of the lungs, were fixed for 24 h in 10% neutral buffered formalin (Applied Biosystems, Waltham, MA, USA), dehydrated, embedded in paraffin (Carl Roth, Karlsruhe, Germany), and sectioned at several 4–5 µm thick tissue sections. Heart sections were stained with the hematoxylin and eosin (H&E) fast staining kit (Carl Roth, Karlsruhe, Germany) to evaluate the extent of myocardial injury, and the percentage of myocardium infiltrated with cells at 65 days pi. At least 5 views of the heart sections from different sectioning levels were selected in a blinded manner and then captured (at 13.5× magnification) under white light using a Nikon SMZ18 microscope (Nikon, Tokyo, Japan). The percentage of nuclei (stained blue) area compared to the entire area of heart tissue (pink) was calculated. Sirius Red staining was performed to observe collagen deposition and to evaluate fibrosis severity in the heart. The whole cardiac cross-sections were captured at 1× magnification under white light and analyzed to reveal the percentage of red collagen area to the area of the entire tissue. Siderophages (HF cells) per lung section area (cells/mm^2^) were determined by Prussian blue staining. All evaluations of histologic sections were performed using the ImageJ (version 1.8.0_112; National Institutes of Health, Bethesda, MD, USA) program.

### 2.6. Quantitative RT-PCR

Total RNA was extracted from the frozen apical LV region of the heart, according to the manufacturer’s protocol using TRIzol Reagent (Invitrogen, Carlsbad, CA, USA). The purified RNA pellets were dissolved in 0.1 mM EDTA (Thermo Fisher Scientific, Waltham, MA, USA), and their concentrations and purity were determined by a NanoPhotometer P300 (Implen, Inc., Westlake Village, CA, USA) reading at 260 and 280 nm. cDNA was synthesized using a High-Capacity cDNA Reverse Transcription Kit (4368814) (Applied Biosystems, Waltham, MA, USA) according to the manufacturer’s instructions; resultant cDNA was stored at −80 °C until analysis. RT-PCR reactions were performed with the Power SYBR Green PCR Master Mix (2x) (Applied Biosystems, Waltham, MA, USA) in 96-well PCR plates (Thermo Fisher Scientific, Waltham, MA, USA) using a Bio-Rad CFX96 Thermocycler (Bio-rad, Hercules, CA, USA). The used oligonucleotide primer sequences for target genes were synthesized by Metabion International AG (Planegg/Steinkirchen, Germany) and are listed in Table S2, Supplementary Materials; the final concentration of 200 nM in the reaction was used. Reactions of the total volume of 20 μL and cDNA at the final concentration of 10 ng were performed in duplicate. The initial step started at 95 °C for 10 min and proceeded with 40 cycles of 95 °C for 15 s, then 60 °C for 1 min for gene amplification. Quantitative PCR data were analyzed using the ∆∆Ct method. The mRNA expression level of each gene in each sample was quantified relative to that of the *18S* rRNA in the same sample. Values were normalized to that of the corresponding sham mice to represent a fold change. 

### 2.7. Protein Isolation and Western Blot Analysis

Total protein extracts were prepared by homogenizing heart tissue and suspending it in ice-cold lysis buffer (8 M urea (AppliChem, Darmstadt, Germany); 2 M thiourea (Merck, Darmstadt, Germany); 100 mM DTT (Thermo Fisher Scientific Baltics, Vilnius, Lithuania); 3% SDS (*w*/*v*) (Lach-Ner, Neratovice, Czech Republic); 0.05 M Tris-HCl (Carl Roth, Karlsruhe, Germany), pH = 6.8), supplemented with protease and phosphatase inhibitors cocktail (10 μg/mL aprotinin (Abcam, Cambridge, UK), 1 mM PMSF (Roche Diagnostics, Mannheim, Germany), and 1 mM Na_3_VO_4_ (AppliChem, Darmstadt, Germany)). The supernatant was separated by centrifugation at 12,000× *g* for 15 min at 4 °C. Protein samples were mixed with 4x sample buffer (25% 1 M Tris-HCl (*v*/*v*), pH = 6.8; 40% of 100% glycerol (AppliChem, Darmstadt, Germany); 8% SDS; 4% 14.3 M β-mercaptoethanol (Carl Roth, Karlsruhe, Germany); 0.1% bromophenol blue (Sigma-Aldrich, Darmstadt, Germany)), denatured at 95 °C for 10 min, then cooled and stored at −20 °C or −80 °C until analysis. Equal amounts of protein from each sample were separated by 12% SDS-PAGE gel and transferred onto a nitrocellulose membrane (Thermo Scientific, Waltham, MA, USA) using semi-dry electrophoretic conditions. After, membranes were blocked for 1 h at room temperature in TBST (50 mM Tris-HCl, pH = 8.0; 150 mM NaCl (Carl Roth, Karlsruhe, Germany); and 0.1% Tween-20 (Carl Roth, Karlsruhe, Germany)) containing 3% bovine serum albumin (PAN-Biotech, Aidenbach, Aidenbach, Germany). Then, the blots were incubated with primary antibodies (Table S3, Supplementary Materials) overnight at 4 °C. The following day, blots were rinsed in TBST 3 times, for 5 min each, and incubated for 1 h at room temperature with HRP-conjugated secondary antibodies (at 1:10,000 dilution) (Invitrogen, Carlsbad, CA, USA). After being rinsed in TBST 3 times, for 5 min each, target proteins were detected using ECL reagent (Thermo Scientific, Waltham, MA, USA). Heart samples from unimmunized WT mice (*n* = 3) of the same age were pooled and used for protein signal standardization on the membranes; GAPDH protein was used as a loading control. Protein bands were visualized and quantified using the Alliance Q9 imaging system and NineAlliance 4.7 17.00 software (UVITEC, Cambridge, UK).

### 2.8. Statistical Analysis

The main statistical analyses were conducted in R (version 4.2.0; R Core Team, www.r-project.org, accessed on 12 November 2022). The Shapiro–Wilk test was applied to assess the normality of data. The Kruskal–Wallis nonparametric test with Dunn’s post hoc was used to analyze ordinal data. The equality of variance of normally distributed results was evaluated with Levene’s test. Due to the heteroscedasticity of our data, robust one-way and two-way analysis of variance (ANOVA) (“WRS2” package in R: functions *t2way*, *t1way*, and post hoc linear contrasts (lincon) with 10% trimming of the means) were performed. Analysis of covariance (ANCOVA), followed by estimated marginal means (EMMs) post hoc test, were used to determine the differences in the means between α-MHC-immunized animals groups at DCM phase (65 days pi); the covariate used is specified in the legends of figures. Principal component analysis (PCA) was performed to simplify the complexity of high-dimensional data while retaining trends and patterns between the groups. A *p*-value of ≤ 0.05 was considered statistically significant. Statistically significant differences in graphs are noted with *, #, $ signs: *,# (*p* < 0.05), **, ## (*p* < 0.01) and ***, ### (*p* < 0.001), $ (*p* < 0.05). Simple linear regression was performed to identify relationships among global and micro markers of disease progression. The coefficient of determination (R^2^) was used to evaluate the strength of the linear relationship. Results were considered statistically significant when the observed *p*-value was < 0.05. GraphPad Prism v6.0 (La Jolla, CA, USA) and R programs were used for the preparation of graphs. The name of the statistical test used, and the exact presentation form of the data are stated in the legends of figures alongside replicate (*n*) numbers.

## 3. Results

### 3.1. EAM Leads to HF in ANKRD1-Deficient Mice

**Figure 1:**
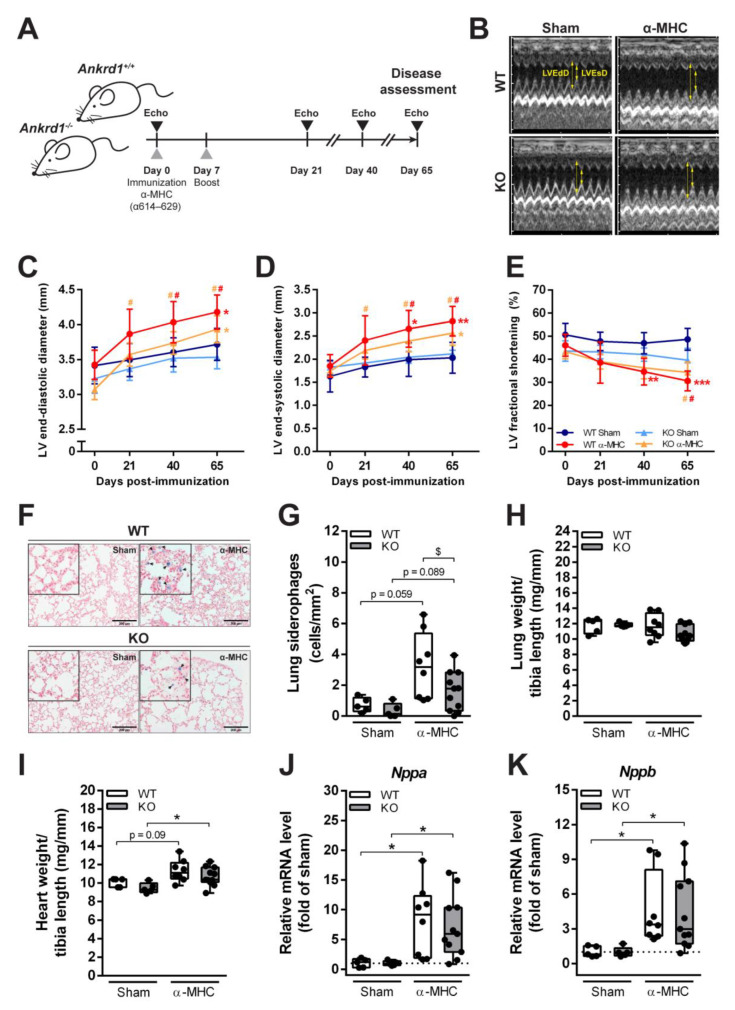
Development of DCM and HF in response to EAM induction. (**A**) Experimental design scheme. (**B**) Representative images of M-mode echocardiograms at the short parasternal axis of sham- and α-MHC-immunized WT and *Ankrd1* KO mice hearts 65 days pi. Yellow arrows indicate LVEdD—left ventricular end-diastolic dimension and LVEsD—left ventricular end-systolic dimension. (**C–E**) Echocardiographic parameters of mice hearts during the 65-day time course. LV—left ventricular. Data represented as mean ± 95% CI. *—marks statistically significant differences from respective sham, #—differences from day 0, the color of the mark corresponds to the colors of the groups. *,# (*p* < 0.05), **,## (*p* < 0.01), *** (*p* < 0.001)—marks statistically significant differences, by robust ANOVA followed by Lincon post hoc. (**F**) Representative Prussian blue staining of lung sections (scale bar – 200 µm) showing HF cells—siderophages (blue, denoted with black arrows) in the lung parenchyma of sham- and α-MHC-immunized mice and (**G**) quantitative analysis of their distribution in the lung tissue. (**H**) Lung weight to tibia length ratios. (**I**) Heart weight to tibia length ratios. (**J**,**K**) Quantitative RT-PCR analysis of *Nppa* and *Nppb* transcripts levels, data were normalized to corresponding *18S* rRNA levels and expressed as the fold change versus the corresponding sham control; the dotted line represents sham levels. WT-Sham (*n* = 5), WT-α-MHC (*n* = 8), KO-Sham (*n* = 5) and KO-α-MHC (*n* = 11). Data represented as box-plots depicting median ± IQR, black dots denote individual animals. * (*p* < 0.05), ** (*p* < 0.01), *** (*p* < 0.001)—marks statistically significant differences, by robust ANOVA followed by Lincon post hoc. $ (*p* < 0.05)—marks statistically significant differences between α-MHC-immunized groups, by ANCOVA (LVEdD was used as a covariate) followed by EMMs post hoc.

Both *Ankrd1^+/+^* (WT) and *Ankrd1^−/−^* (KO) mice were immunized with cardiac α-MHC peptide and EAM/DCM progression was evaluated as shown in Figure 1A. Echocardiographic evaluation of LV chamber dimensions and systolic heart function were performed on 0, 21, 40 days pi and the EAM-induced DCM stage 65 days pi (Figure 1B–E; Figure S3, Table S4, Supplementary Materials). Echocardiograms showed a significant increase in LV end-diastolic (1.1-fold over sham) and end-systolic (1.4-fold over sham) dimensions 65 days pi in α-MHC-immunized WT mice group (Figure 1B–D). Moreover, LV fractional shortening, an index of heart contractility, was reduced (1.6-fold below sham) (Figure 1E) in the same group. These observations are in good agreement with the pattern of post-MC cardiac remodeling leading to DCM development. *Ankrd1* KO mice immunized with α-MHC showed similar kinetics of LV dilatation (LV end-diastolic 1.1-fold over sham, LV end-systolic 1.2-fold over sham) at 65 days pi; nevertheless, they demonstrated a preserved contractility of the heart compared to α-MHC-immunized WT animals (Figure 1E). Siderophages (HF cells) are pulmonary macrophages that phagocytize erythrocytes leaked from the congested capillaries due to increased pulmonary blood pressure during HF. Hemosiderin accumulates in the cytoplasm of macrophages and stains blue after lung tissue staining with Prussian blue. The presence of siderophages at 65 days pi was already observed in the lung parenchyma, in both WT and *Ankrd1* KO α-MHC-immunized mice groups (Figure 1F,G). However, lower numbers were detected in ANKRD1-deficient mice. Yet, EAM at 65 days pi had no effect on lung weight (index of lung congestion) (Figure 1H). In addition, an increase in heart mass (Figure 1I) and higher mRNA expression of *Nppa* (natriuretic peptide A) and *Nppb* (natriuretic peptide B) (markers of heart stress) were determined (Figure 1J,K) in both WT and *Ankrd1* KO α-MHC-immunized mice groups (compared to respective sham). Taken together, these data suggest that both WT and *Ankrd1* KO α-MHC-immunized mice 65 days after EAM induction developed HF.

### 3.2. Loss of ANKRD1 Is Beneficial in the EAM-Induced DCM

**Figure 2:**
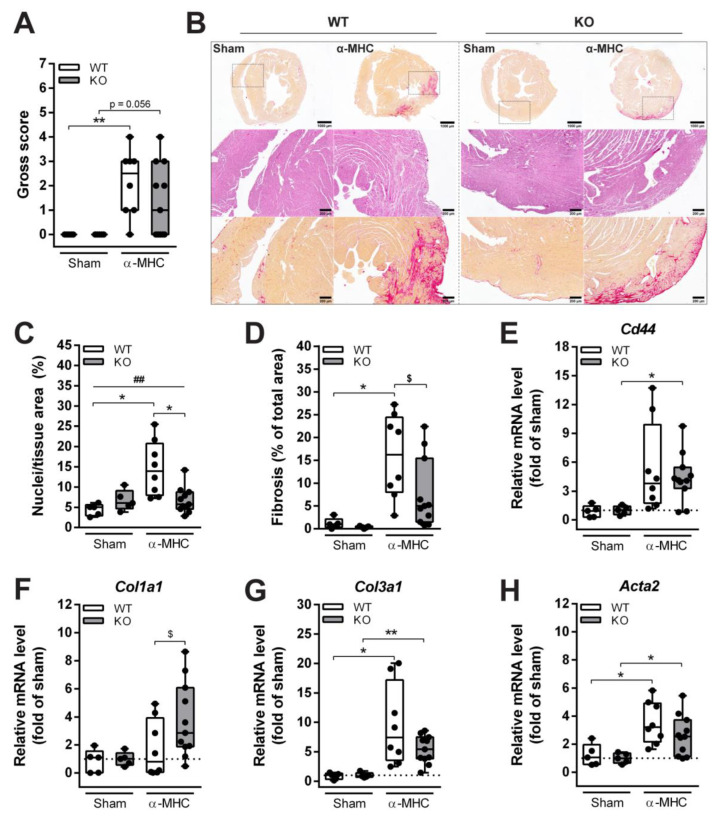
Histological and biochemical characterization of cardiac remodeling induced by EAM. (**A**) The gross score of the dissected mice hearts 65 days after EAM induction. ** (*p* < 0.01) marks statistically significant differences, by the Kruskal–Wallis test, and differences between groups were determined by Dunn’s post hoc test. (**B**) Representative sections of whole mouse hearts 65 days after EAM induction (upper panel, 1.5× magnification) stained with Sirius red for collagen (red) (scale bar—1000 µm), sections stained with H&E (middle panel, 6× magnification) (scale bar—200 µm) and Sirius red (lower panel, 6× magnification) (scale bar—200 µm). (**C**) Quantitative analysis of infiltrates found in the heart. (**D**) Quantification of heart fibrosis. The mRNA expression of (**E**) *Cd44*, (**F**) *Col1a1*, (**G**) *Col3a1*, and (**H**) *Acta2*, data were normalized to corresponding *18S* rRNA levels and presented as the fold change versus the corresponding sham control; the dotted line marks sham levels. WT-Sham (*n* = 5), WT-α-MHC (*n* = 8), KO-Sham (*n* = 5) and KO-α-MHC (*n* = 11). Data represented as median ± IQR. Black dots denote individual animals. * (*p* < 0.05), ** (*p* < 0.01) marks statistically significant differences and ## (*p* < 0.01) marks statistically significant differences in interaction between genotype and immunization, by robust ANOVA followed by Lincon post hoc. $ (*p* < 0.05) marks statistically significant differences between α-MHC-immunized groups, by ANCOVA (Gross score was used as a covariate) followed by EMMs post hoc.

Both WT and *Ankrd1* KO α-MHC-immunized animals exhibited similar dynamics and extent of LV dilatation, and also a pronounced upregulation of cardiac stress markers (such as *Nppa* and *Nppb*) was induced. Yet, cardiac dysfunction was milder in *Ankrd1* KO animals, as demonstrated by a lower number of siderophages found, and preserved systolic function 65 days pi. Thus, further analysis of post-MC DCM progression was performed. At 65 days pi, dissected mice hearts were grossly scored and a similar severity of cardiac damage was registered in both α-MHC-immunized animal groups (Figure 2A). In addition, analyses of the hematoxylin and eosin (H&E) stained cardiac sections have not shown any interstitial cellular infiltration (determined by the area of nuclei per tissue area (%)) in *Ankrd1* KO-α-MHC hearts (did not exceed sham level), whereas infiltration in α-MHC-immunized WT hearts was higher (3.3-fold over sham) (Figure 2B (middle panel) and C). These results were accompanied by cardiac fibrosis evaluation. Sirius red staining of sham-immunized mice hearts showed only mild fibrosis, evenly distributed through the myocardium, and physiologically more intensive around the vessels (Figure 2B (upper and lower panels) and D). While severe interstitial fibrosis, mostly located in the outer part of the myocardium, was found in α-MHC-immunized WT and *Ankrd1* KO mice groups, the extent of fibrosis in KO mice was (2-fold) lower than in WT mice (Figure 2B (upper and lower panels) and D). Cardiac fibrosis is a process of pathological cardiac remodeling, which is mediated through the induction of various genes. Thus, a quantitative mRNA level analysis of extracellular matrix remodeling-associated genes - *Col1a1* (collagen 1a1), *Col3a1* (collagen 3a1), *Cd44* and *Acta2* (α-smooth muscle actin) was conducted. The mRNA levels of these genes tended to increase in both WT and *Ankrd1* KO α-MHC-immunized mice compared to the corresponding sham mice (Figure 2E–H). Furthermore, *Col1a1* levels were more (2.2-fold) upregulated in *Ankrd1* KO-α-MHC mice (Figure 2F). To summarize, the more severe cardiac lesions observed in WT animals hearts suggest that ANKRD1 deficiency can blunt cardiac damage during MC-induced cardiac remodeling.

### 3.3. ANKRD1 - Deficiency Affects Overall Levels of MAPK/AP1 and the Response of Mechanosensing-Related Proteins in Post-EAM DCM

**Figure 3:**
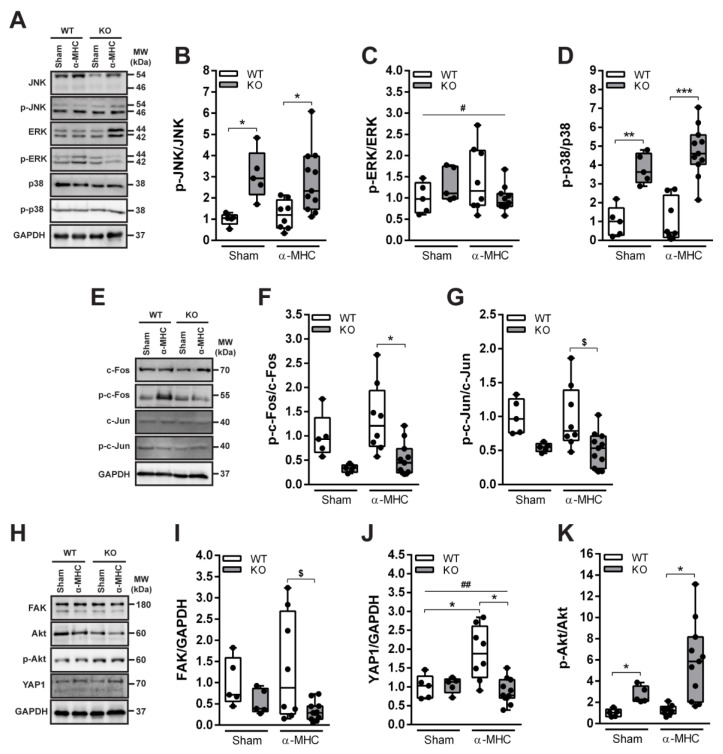
Western blot analysis of the MAPK/AP1 and mechanosensing-related proteins (FAK, Akt, and YAP1) levels in mice 65 days pi (post-MC DCM stage). (**A**) Representative immunoblots and (**B**–**D**) quantification analysis of MAPK’s JNK, ERK, and p38 proteins’ activation. (**E**) Representative Western blot and (**F**,**G**) quantitative densitometric analysis of AP1 components c-Fos and c-Jun proteins phosphorylation. (**H**) Representative immunoblots and (**I**–**K**) quantification of FAK, Akt and YAP1 proteins. WT-Sham (*n* = 5), WT-α-MHC (*n* = 8), KO-Sham (*n* = 5) and KO-α-MHC (*n* = 11). GAPDH served as a loading control. Data are represented as the median ± IQR, black dots denote individual animals. * (*p* < 0.05), ** (*p* < 0.01), *** (*p* < 0.001) marks statistically significant differences, # (*p* < 0.05), ## (*p* < 0.01) marks statistically significant differences in interaction between genotype and immunization, by robust ANOVA followed by Lincon post hoc. $ (*p* < 0.05) marks statistically significant differences between α-MHC-immunized groups, by ANCOVA (Gross score was used as a covariate) followed by EMMs post hoc.

Mitogen-activated protein kinases (MAPKs) are involved in the regulation of various cellular responses, and are considered to be intimately involved in cardiac remodeling during pathological conditions [28]. Thus, the phosphorylation status of three main members of the MAPK family—extracellular signal-regulated kinase (ERK), c-Jun N-terminal kinase (JNK), and p38 kinase in mice hearts at the post-MC DCM stage (65 days pi) were evaluated. Immunization had no effect on total and phosphoform levels of JNK, ERK, and p38 (Figure 3A–D). However, ANKRD1-deficient mice had increased levels of phosphorylated JNK (3.0-fold over WT mice) and p38 (3.8-fold over WT mice) kinases (Figure 3B,D). Whereas, genotype had no impact on the ERK phosphorylation (Figure 3C). 

MAPK signaling converges into early immediate activation of several transcription factors including activator protein-1 (AP-1) [28]. Thus, the activations of AP-1 components c-Fos and c-Jun were evaluated. Similarly to MAPKs, no changes in c-Fos and c-Jun proteins phosphorylation were found among the α-MHC-immunized mice and corresponding sham groups (Figure 3E–G). In addition, phospho-levels of c-Fos (2.8-fold lower than WT mice) and c-Jun (1.9-fold lower than WT mice) were reduced in ANKRD1-deficient animals (Figure 3F,G). These results suggest that on 65 days pi, MAPK and c-Fos, c-Jun did not participate in post-MC cardiac remodeling leading to DCM. However, we see that ANKRD1-deficient mice differ substantially in their activation of MAPK and AP-1 (c-Fos, c-Jun), and this may have an impact on the overall outcome of the disease seen in *Ankrd1* KO animals. 

The increased levels of focal adhesion kinase (FAK) and yes-associated protein 1 (YAP1) are closely associated with fibrosis and extracellular matrix (ECM) remodeling [29,30] during HF [31,32]. These two proteins also are known to promote the activation of protein kinase B (Akt) [33,34], which is a major regulator of cellular responses during cardiac stress [35]. Thus, the levels of FAK, YAP1, and Akt proteins were evaluated in animal hearts at post-MC DCM stage (65 days pi) (Figure 3H–K). Our results show that α-MHC-immunized WT animals had significantly higher total levels of FAK and YAP1 (4-fold and 2.2-fold, respectively) than α-MHC-*Ankrd1* KO (Figure 3I,J). Furthermore, ANKRD1-deficient mice had increased (4-fold) Akt phosphorylation compared to WT animals and a tendency of higher (4.3-fold over sham) Akt activation in hearts 65 days pi with α-MHC (Figure 3K). Altogether, these results suggest that during post-MC DCM (65 days pi) FAK and YAP1 proteins did not participate in ANKRD1-deficient mice’s response to EAM, whilst the propensity of Akt phosphorylation in these mice suggests its possible involvement.

### 3.4. ANKRD1 Is Not a Major HF Mediator in Post-MC DCM

**Figure 4:**
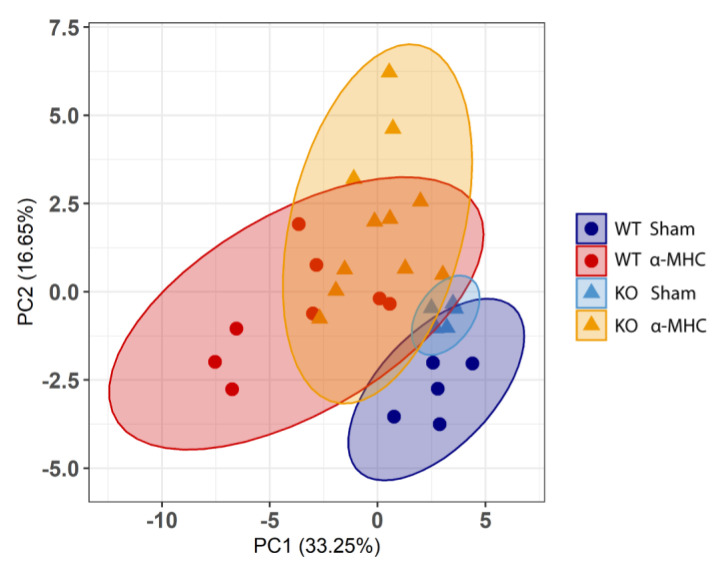
Principal component analysis (PCA) of overall experimental data. Plot shows distribution of WT-Sham (*n* = 5) (blue, dot), WT-α-MHC (*n* = 8) (red, dot), KO-Sham (*n* = 5) (light blue, triangle) and KO-α-MHC (*n* = 11) (yellow, triangle) across the first two principal components (32 features per PCA were used). The explained variance per component (PC1, PC2) (%) is indicated in the panel. The 95% confidence ellipses are represented by the corresponding color of the animal group.

To investigate the overall differences between WT and *Ankrd1* KO mice during post-MC cardiac remodeling, a principal component analysis (PCA) based on (32) features tracked during this study was performed (Table S5, Supplementary Materials). Score plots were applied to visualize the distribution patterns of animals and their groups. The two-dimensional scatter plots of the WT and *Ankrd1* KO mice were defined by the first and second principal components PC1 and PC2. PCA analysis (Figure 4) (PC1 [33.25%]; PC2 [16.65%]; both explained 50% of the total variance) revealed distant yet overlapping clusters. Clusters representing both sham groups showed less scattering compared with α-MHC immunized groups and did not have overlapping points between them. In the case of α-MHC groups, the scattering between both genotypes was overlapping; however, the patterns were distinctive. This PCA analysis summarizes previous observations showing that both WT and *Ankrd1* KO mice had similar, yet different, responses to EAM, and this implies that ANKRD1 is not a major HF mediator during the MC-induced DCM development.

## 4. Discussion

Given the evidence of ANKRD1 linkage to human cardiovascular diseases, and its upregulation during various cardiac stress conditions [14,16,36,37], we evaluated the cardiac consequences of global ANKRD1 loss upon EAM-induced DCM [23,26,27] utilizing global *Ankrd1* KO mice.

Immunization of both WT and global *Ankrd1* KO mice—with cardiac α-MHC induced EAM, sequentially leading to DCM with progressive dilatation of LV along with deteriorating systolic function—was assessed by fractional shortening. Histologically, immunization caused cellular infiltration and fibrosis located mostly in the outer layers of the myocardium, accompanied by accumulation of siderophages, otherwise known as HF cells in lung parenchyma. Moreover, mRNA profiling of α-MHC immunized WT and *Ankrd1* KO mice hearts revealed upregulation of cardiac stress and remodeling markers (*Nppa*, *Nppb*, *Col1a1*, *Col3a1*, *Cd44* and *Acta2*). Taken together, our used murine EAM-induced DCM model corresponded with the key features of post-MC cardiac remodeling, leading to DCM similar to that observed in failing DCM human hearts. Nevertheless, *Ankrd1* KO animals exhibited a lower number of siderophages, less fibrosis, and better preservation of cardiac contractility compared to WT animals. Altogether, these findings indicate that loss of ANKRD1 mitigates cardiac damage and remodeling in MC-induced DCM.

**Figure 5:**
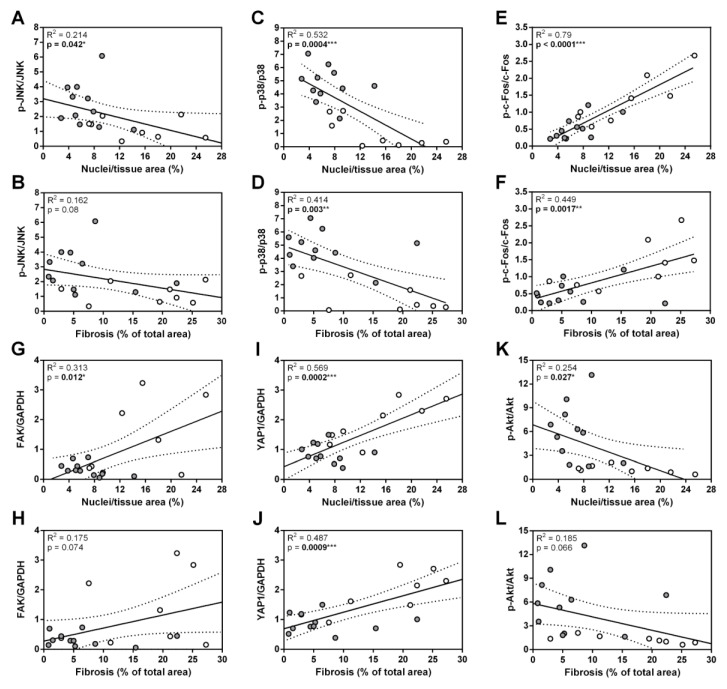
Summarized interactions between heart damage and the levels of proteins found in mice during post-MC DCM (65 days pi). Linear regressions of (**A**) p-JNK/JNK, (**C**) p-p38/p38, (**E**) p-c-Fos/c-Fos, (**G**) FAK/GAPDH, (**I**) YAP1/GAPDH, (**K**) p-Akt/Akt on Nuclei/tissue area (cellular infiltration). Linear regressions of (**B**) p-JNK/JNK, (**D**) p-p38/p38, (**F**) p-c-Fos/c-Fos, (**H**) FAK/GAPDH, (**J**) YAP1/GAPDH, (**L**) p-Akt/Akt on Fibrosis. The WT-α-MHC (white dots) and *Ankrd1* KO- α-MHC (gray dots) groups were combined. The regression line is represented within a 95% confidence interval. The coefficients of determination (R^2^) from linear regression analysis and *p*-values are shown on the graphs. * (*p* < 0.05), ** (*p* < 0.01), *** (*p* < 0.001) marks statistically significant correlations.

Three main members of the MAPK protein family ERK, JNK, and p38 regulate a diverse array of physiological processes in mammalian cells and tissues in response to pathological stimuli [28]. Activation of these kinases was shown in different heart diseases, including MC and DCM [28,38,39,40,41]. ERK, JNK, and p38 can play both protective and deleterious roles in the stressed myocardium, depending on type and severity of cardiac stress [42,43,44]. Furthermore, it is known that ANKRD1 and MAPKs have multiple cross-reacting targets and pathways [22,45,46]. Study of ERK, JNK, and p38 in the hearts of end-stage DCM patients demonstrated that the total ERK, JNK, and p38 levels did not vary from healthy hearts; however, activation of these kinases differed greatly, as ERK was upregulated, and activity of p38 decreased, while phosphorylation of JNK did not change in failing hearts [41]. We observed a similar tendency of MAPK action in WT animals during post-MC DCM (65 days pi) (Figure 3A–D). Interestingly, loss of ANKRD1 increased phosphorylation of JNK and p38 in hearts (Figure 3A,B,D), and it was negatively correlated with cardiac fibrosis and cellular infiltration (Figure 5A–D). These observations suggest that the beneficial effects of ANKRD1 loss during MC-induced DCM development could be mediated via JNK and p38 signaling pathways. 

Canonical MAPK signaling converges into early immediate activation of several transcription factors, including activator protein-1 (AP-1) [28]. c-Fos and c-Jun are members of the AP-1 transcription factor family, and have been previously shown to be upregulated in cardiac cells upon immunological, mechanical, and pharmacological stimuli [47,48,49,50]. c-Fos predominantly mediates maladaptive cardiac remodeling [50], and its increased levels are associated with myocardial lesion severity [49]. Whereas, c-Jun activation promotes expression of sarcomeric proteins, suppresses expression of ECM proteins [48], and is usually associated with better cardiac disease outcome [48]; nevertheless, in failing DCM human hearts, its phosphorylation was not detected [41]. Our results confirm all these previous findings and support c-Fos involvement in pathological processes during post-MC cardiac remodeling leading to DCM in α-MHC-immunized WT animals, as the levels of the phosphorylated form were positively correlated with the levels of fibrosis and infiltrate found in hearts (Figure 5E,F). Whereas, the loss of ANKRD1 resulted in significantly lower phosphorylated forms of c-Fos and c-Jun compared to WT animals (Figure 3E–G). Thus, the lower activation of c-Fos found in *Ankrd1* KO animals may be related to their milder response to EAM, and it implies the link between ANKRD1 and AP-1 (c-Fos, c-Jun) activation during post-MC DCM.

FAK is one of the first molecules recruited to focal adhesions in response to various external stimuli [51], and it relays them into the cellular response through various mechanotransducers e.g. YAP1 [52]. Activated YAP1 is translocated to the nucleus where it promotes the expression of genes implicated in cardiac homeostasis and stress response, including *Ankrd1* [53]. FAK and YAP1 proteins were shown to be upregulated during ECM remodeling [31,32] in DCM [32,54] and other non-ischemic HF conditions [31,53,55]. Furthermore, the FAK—YAP signaling axis is a mechanosensitive mediator of cardiac fibroblast activation [31,56]. In addition, during stretch/stress conditions, FAK and YAP1 are known to promote the activation of Akt [53,56], which is known for its cardioprotective role and ability to enhance the function of failing cardiomyocytes [57,58]. It can also regulate *Ankrd1* expression through the Akt/NF-kB pathway [59]. In good agreement with published data, we found FAK and YAP1 upregulation in α-MHC-immunized WT animals at the post-MC DCM stage (65 days pi) (Figure 3H–J) and their levels positively correlated with cardiac fibrosis and infiltration (Figure 5G–J). While, the loss of ANKRD1 led to increased phospho-Akt levels in the hearts (Figure 3H,K), which negatively correlated with cardiac fibrosis and infiltration (Figure 5K,L). Speculatively, *Ankrd1* might be involved in the regulation of FAK, YAP1, and Akt positive/negative feedback loops during post-MC cardiac remodeling, leading to DCM.

The genetic ablation of *Ankrd1* in MLP (muscle LIM protein) knockout mice (the genetic model of DCM) directly inhibits maladaptive protein kinase C alpha (PKCα) (negatively regulated by MLP) signalosome formation (regulating cardiac contractility and propensity toward HF), thereby preventing morphological, functional, and molecular development of the DCM phenotype [21]. Furthermore, the knockdown of *Ankrd1* attenuated phenylephrine-induced cardiac hypertrophy by inhibiting ANKRD1/ERK/GATA4 (involved in regulating cardiomyocyte growth) complex formation in the sarcomere and its nuclear translocation [22]. Our results, however, show that the EAM-induced DCM was comparable between *Ankrd1* KO and WT mice, and therefore argue against a major role of ANKRD1 in MC/DCM modulation. These results are consistent with ANKRD1-deficient mice’s response to TAC-induced pressure overload, where lack of *Ankrd1* had no impact on cardiac hypertrophy development [22]. Thus, EAM-induced DCM may evoke a more global response by recruiting diverse sarcomere signaling complexes, compensating for the loss of ANKRD1 signaling in the *Ankrd1* KO mouse.

*ANKRD1* is a ubiquitously expressed gene found in numerous tissues, including liver, lungs, and skin [60]. EAM is a complex model that engages different cell types and organ systems. Thus, observed changes between WT and global *Ankrd1* KO animals might be mediated by other types of cells than cardiomyocytes. The global genetic ablation of *Ankrd1* could affect the response to EAM via modulation of the immune system and non-cardiomyocyte orchestrated cardiac tissue remodeling. Firstly, the observed differences could be related to ANKRD1 functions in immune cells, as ANKRD1 has been reported to modulate and correlate with inflammatory states [61,62,63]. Secondly, ANKRD1 could influence remodeling-associated fibrosis, as a loss of ANKRD1 impairs contraction and enhances necrosis of ischemic wounds via changes in dermal fibroblast physiology [64]. Thus, it remains unclear whether the lack of ANKRD1 in cardiomyocytes or other types of cells causes the positive effect of global *Ankrd1* ablation during MC-induced DCM. Future studies are needed to address the more detailed underlying mechanisms.

Altogether, our observations suggest that ANKRD1 does not play a major role during post-MC cardiac remodeling leading to DCM. However, the genetic ablation of *Ankrd1* blunted the cardiac damage/remodeling and preserved heart function. 

## Data Availability

The datasets generated during and/or analyzed during the current study are available from the corresponding author upon reasonable request.

## Supplementary Materials

The following supporting information can be downloaded at: https://www.mdpi.com/article/10.3390/biom12121898/s1, Figure S1: Validation of ANKRD1 loss in *Ankrd1*^−/−^ (KO) mice; Figure S2: Body weight changes in mice after EAM induction; Figure S3: Representative original uncropped echocardiographic images of Sham and α-MHC mice prior immunization and at 65 day post-immunization used for the preparation of Figure 1; Figure S4: Images of original uncropped Western blots used for the preparation of Figure 3; Table S1: Primers used in PCR for mice genotyping; Table S2: Primer sequences of genes analyzed by real-time PCR; Table S3: List of antibodies used in Western blotting; Table S4: Echocardiographic analysis of WT and *Ankrd1* KO mice hearts before and after EAM induction; Table S5: The list of features used for PCA.
