# Supplementary material for: Genetic Ablation of Ankrd1 Mitigates Cardiac Damage during Experimental Autoimmune Myocarditis in Mice
Source: Biomolecules. 2022 Dec 18;12(12):1898. doi: 10.3390/biom12121898 (PMC9775225; doi:10.3390/biom12121898)
Supplement: Supplementary file 1 [file biomolecules-12-01898-s001.zip › biomolecules-2062312-supplementary.pdf]

Supplementary Material

Figure S1. Validation of ANKRD1 loss in *Ankrd1*$^{-/-}$ (KO) mice. (**A**) PCR products from mouse tail DNA samples which were amplified using primers listed in Table S1 (Supplementary Materials). M – low range DNA marker, NTC – no tissue control, Ctrl$^{+/+}$ - DNA from WT BALB/c mice tail, Ctrl$^{-/-}$ - DNA from primary F0 generation KO mice. (**B**) Representative Western blot and (**C**) corresponding quantitative densitometric analysis of ANKRD1 in WT (n = 3) and KO (n = 3) mice hearts lysates. GAPDH was used as a loading control. Data are represented as the mean ± 95% CI. **(p < 0.01) – marks statistically significant differences, by 2-tailed *Student's t-test* with unequal variances.

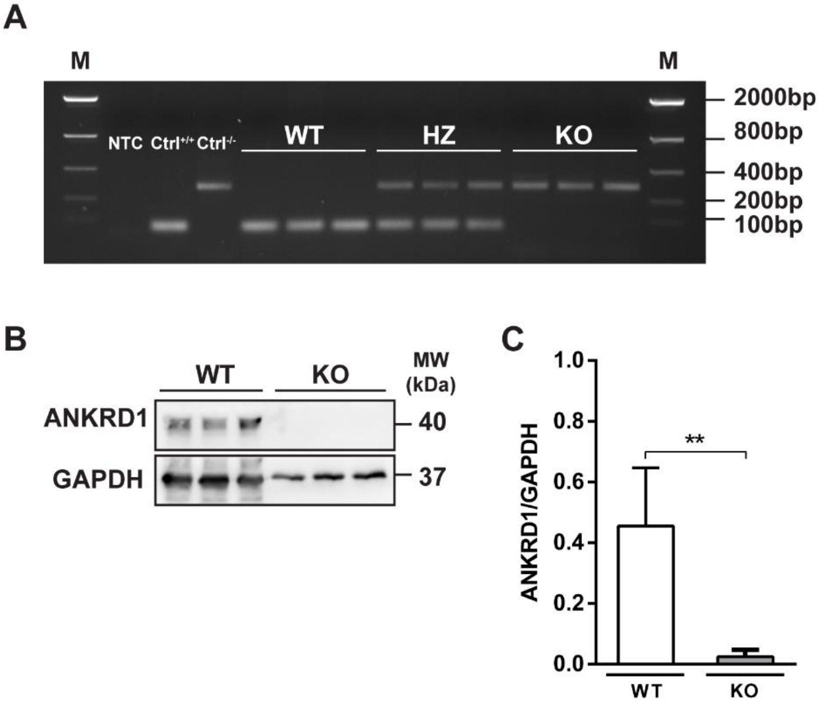

Figure S2. Body weight changes in mice after EAM induction. WT-Sham (n = 5), WT-α-MHC (n = 8), KO-Sham (n = 5) and KO-α-MHC (n = 11). The values are represented as mean ± SEM.

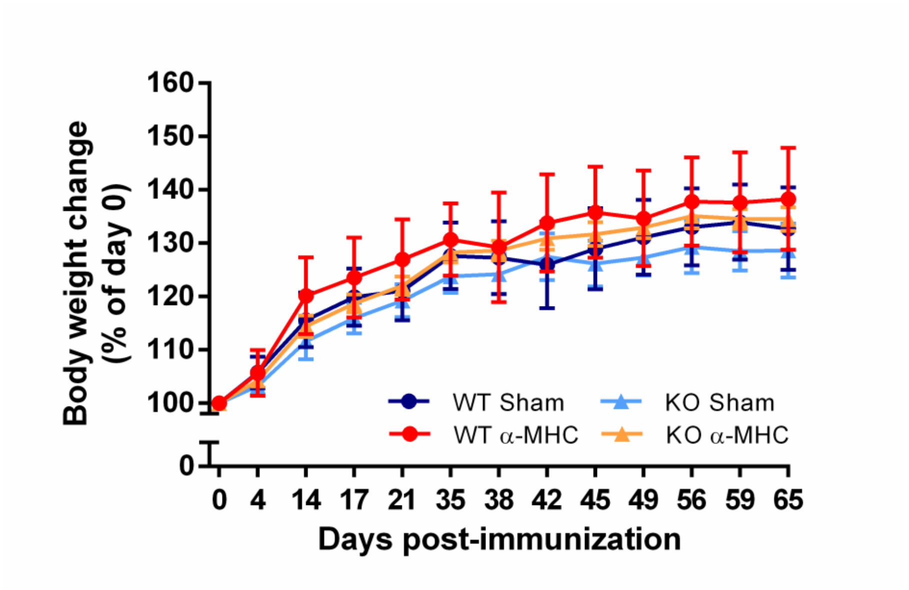

Figure S3. Representative original uncropped echocardiographic images of Sham and α-MHC mice prior immunization and at 65 day post-immunization used for the preparation of Figure 1.

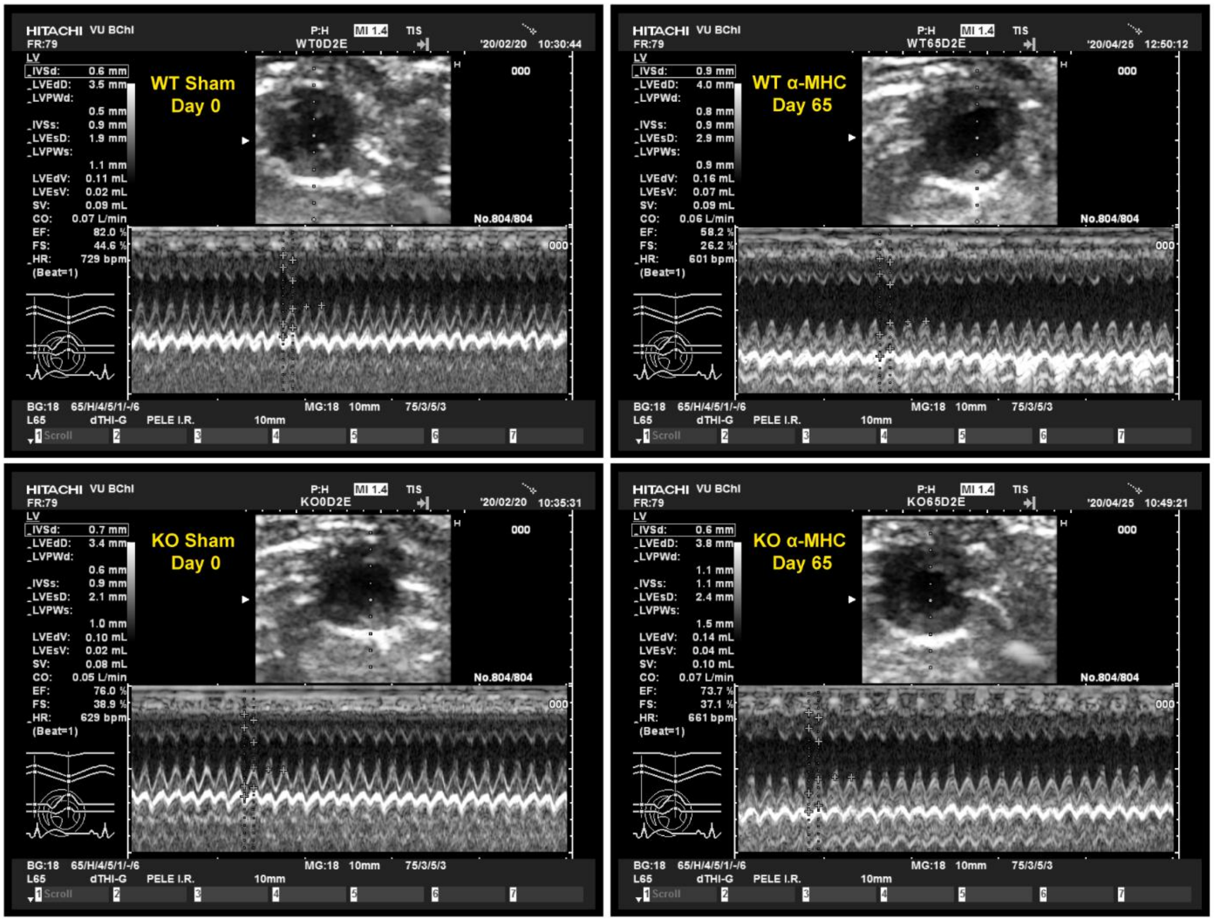

Figure S4. Images of original uncropped Western blots used for the preparation of Figure 3.

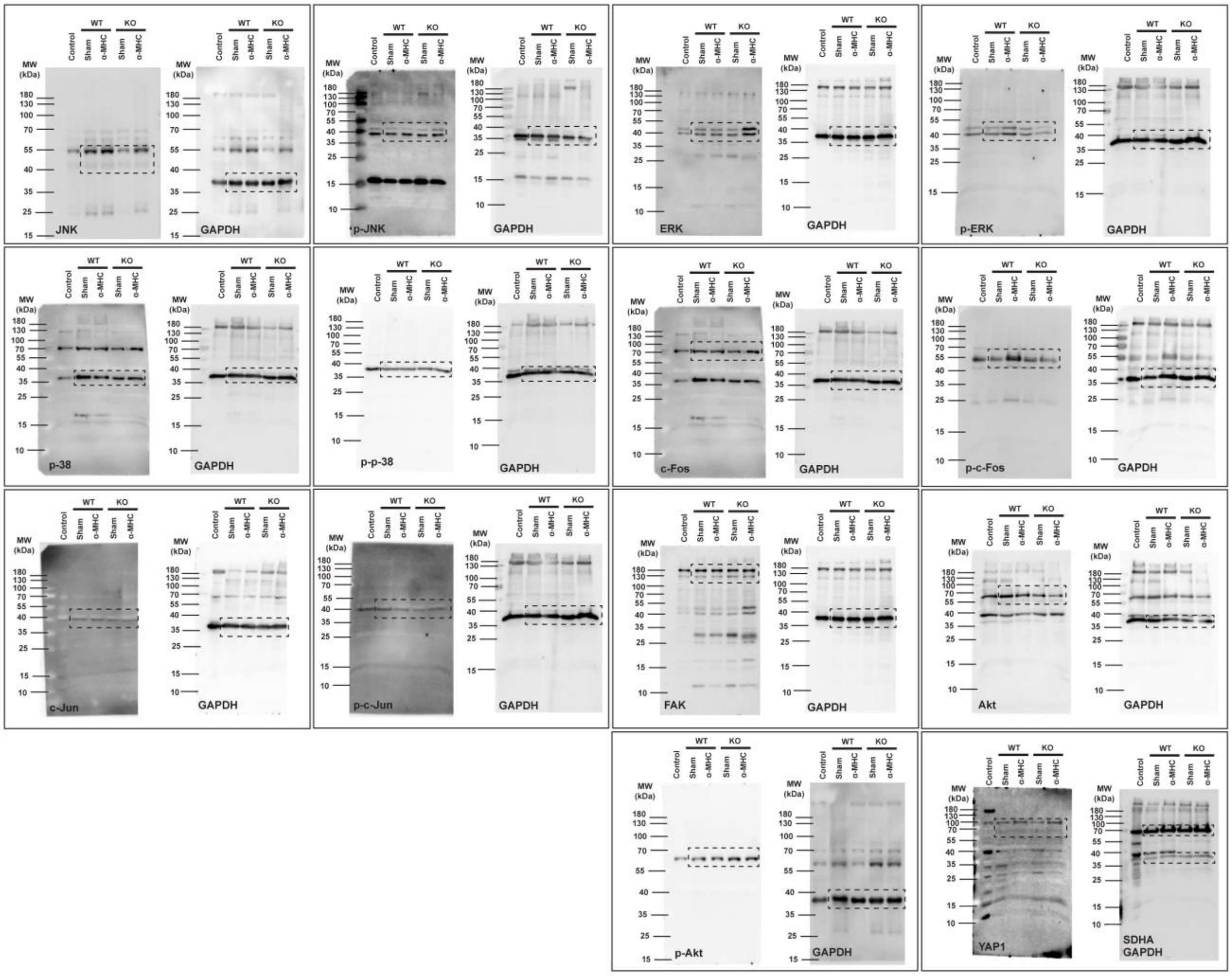

Table S1. Primers used in PCR for mice genotyping.

Primer	Primer Sequences	Length	WT	HZ	KO
NeoFwd	FW: 5'-TCATTCTCAGTATTGTTTTGCC- 3'	268 bp	-	+	+
SD	RV: 5'-TCACTAGAGGATATTTTAAC- 3'				
TUF	FW: 5'-AAGGCAGGCACCTGGGATAC- 3'	68 bp	+	+	-
TUR	RV: 5' -CAGCGACAGCCACCTTCAG- 3'				

Table S2. Primer sequences of genes analyzed by real-time PCR.

Gene	Primers
Col1a1	FW: 5'- TCACCAAACTCAGAAGATGTAGGA -3'<br>RV: 5'- GACCAGGAGGACCAGGAAG -3'
Col3a1	FW: 5'- ACAGCAGTCCAACGTAGATGAAT -3'<br>RV: 5'- TCACAGATTATGTCATCGCAAAG -3'
Nppa	FW: 5'- CCCCGACCCACGCCAGCAT -3'<br>RV: 5'- TGCGGCCCCTGCTTCCTCA -3'
Nppb	FW: 5'- AAGCTGCTGGAGCTGATAAGA -3'<br>RV: 5'- GTTACAGCCCAAACGACTGAC -3'
Ankrd1	FW: 5'- GCATTTCCCAACAGAAGAGC -3'<br>RV: 5'- AAGTCTTGCTCCCCCAAAAT -3'
Acta	FW: 5'- TTGCTGACAGGATGCAGAAGGAGA -3'<br>RV: 5'- ATCTGCTGGAAGGTAGACAGCGAA -3'
Cd44	FW: 5'- ATCAGCAGATCGATTTGAATGTAA -3'<br>RV: 5'- CATTTCCTTCTATGAACCCATACC -3'
18S	FW: 5'- GGAAGGGCACCACCAGGAGT -3'<br>RV: 5'- TGCAGCCCCGGACATCTAAG -3'

Table S3. List of antibodies used in Western blotting.

Antibody name	Company	Dilution
ANKRD1	(3119) Myomedix, Neckargemünd, Germany	1:500
JNK	(9258s) Cell Signaling, Danvers, MA, USA	1:1000
p-JNK	(700031) Thermo Scientific, Waltham, MA, USA	1:1000
ERK	(ab184699) Abcam, Cambridge, UK	1:1000
p-ERK	(sc-16982) Santa Cruz Biotechnology, Dallas, TX	1:500
p38	(331300) Thermo Scientific, Waltham, MA, USA	1:1000
p-p38	(MA5-15177) Thermo Scientific, Waltham, MA, USA	1:1000
c-Fos	(MA536080) Thermo Scientific, Waltham, MA, USA	1:2000
p-c-Fos	(PA5-105995) Invitrogen, Carlsbad, CA, USA	1:1000
c-Jun	(ab40766) Abcam, Cambridge, UK	1:1000
p-c-Jun	(558036) BD Biosciences, San Jose, CA, USA	1:500
FAK	(sc-558) Santa Cruz Biotechnology, Dallas, TX	1:1000
YAP1	(PA5-86396) Invitrogen, Carlsbad, CA, USA	1:1000
Akt	(710005) Invitrogen, Carlsbad, CA, USA	1:500
p-Akt	(4060) Cell Signaling, Danvers, MA, USA	1:1000
GAPDH	(PA1-987) Invitrogen, Carlsbad, CA, USA	1:1000
HRP-Goat- anti Rabbit IgG	(4750) Carl Roth, GmbH, Germany	1:10 000
HRP-Goat anti Mouse IgG	(4759) Carl Roth, GmbH, Germany	1:10 000

Table S4. Echocardiographic analysis of WT and *Ankrd1* KO mice hearts before and after EAM induction.

Group	Days post-immunization	LVEdD (mm) Mean ± 95% CI	LVEsD (mm) Mean ± 95% CI	FS (%) Mean ± 95% CI	HR (bpm) Mean ± 95% CI
**WT Sham (n=5)**	0	3.41 ± 0.26	1.65 ± 0.29	51.77 ± 7.6	589 ± 68
	21	3.49 ± 0.24	1.83 ± 0.21	47.75 ± 3.96	669 ± 55
	40	3.6 ± 0.21	1.99 ± 0.36	46.97 ± 4.59	557 ± 79
	65	3.71 ± 0.23	2.03 ± 0.33	48.6 ± 4.69	628 ± 123
The main effect of measurement time		*F (3, 8.86) = 1.99, p = 0.186, η^2= 0.58*	*F (3, 8.69) = 2.03, p = 0.182, η^2= 0.69*	*F (3, 8.75) = 0.69, p = 0.578, η^2= 0.58*	*F (3, 8.33) = 2.17, p = 0.166, η^2= 0.53*
**WT α-MHC (n=8)**	0	3.42 ± 0.21	1.77 ± 0.31	48.04 ± 9.39	500 ± 75
	21	3.87 ± 0.35	2.4 ± 0.53	38.55 ± 8.94	578 ± 66
	40	4.03 ± 0.29 $^{\#\#}$	2.65 ± 0.39 $^{\#\#}$	34.58 ± 5.77	564 ± 52
	65	4.18 ± 0.24 $^{\#\#\#}$	2.82 ± 0.32 $^{\#\#\#}$	30.58 ± 4.30 $^{\#}$	562 ± 87
The main effect of measurement time		*F (3, 15.31) = 10.79, p = **0.00046**, η^2= 0.69*	*F (3, 15.33) = 10.49, p = **0.00053**, η^2= 0.72*	*F (3, 14.88) = 5.33, p = **0.011**, η^2= 0.75*	*F (3, 15.3) = 1.18, p = 0.348, η^2= 0.35*
**KO Sham (n=5)**	0	3.23 ± 0.21	1.82 ± 0.13	43.52 ± 4.43	602 ± 60
	21	3.37 ± 0.16	1.91 ± 0.06	43.11 ± 2.92	625 ± 90
	40	3.52 ± 0.2	2.04 ± 0.13 $^{\#}$	42.02 ± 2.84	656 ± 55
	65	3.53 ± 0.16	2.11 ± 0.08 $^{\#\#}$	40.18 ± 3.47	640 ± 84
The main effect of measurement time		*F (3, 8.85) = 3.73, p = 0.055, η^2= 0.72*	*F (3, 8.53) = 12.03, p = **0.002**, η^2= 0.86*	*F (3, 8.79) = 1.18, p = 0.373, η^2= 0.56*	*F (3, 8.73) = 1.03, p = 0.425, η^2= 0.35*
**KO α-MHC (n=11)**	0	3.07 ± 0.14	1.74 ± 0.11	43.29± 3.14	582 ± 48
	21	3.57 ± 0.17 $^{\#\#\#}$	2.18 ± 0.18 $^{\#\#}$	38.96 ± 3.19	628 ± 50
	40	3.74 ± 0.16 $^{\#\#\#}$	2.39 ± 0.23 $^{\#\#\#}$	36.24 ± 4.73	674 ± 21 $^{\#}$
	65	3.93 ± 0.19 $^{\#\#\#}$	2.56 ± 0.26 $^{\#\#\#}$	34.25 ± 4.46 $^{\#}$	645 ± 38
The main effect of measurement time		*F (3, 17.48) = 27.88, p < **0.0001**, η^2= 0.88*	*F (3, 17.1) = 17.29, p < **0.0001**, η^2= 0.72*	*F (3, 17.59) = 4.2, p = **0.021**, η^2= 0.6*	*F (3, 16.23) = 4.38, p = **0.019**, η^2= 0.52*
Interaction among groups	0	*F (3, 10.93) = 5.28, p = **0.017**, η^2= 0.71 (WT α-MHC vs. KO α-MHC*)*	*F (3, 11.24) = 0.77, p = 0.531, η^2= 0.51*	*F (3, 11.1) = 2.71, p = 0.095, η^2= 0.66*	*F (3, 11.76) = 2.24, p = 0.136, η^2= 0.64*
	21	*F (3, 12.03) = 3.48, p = 0.05, η^2= 0.85*	*F (3, 10.67) = 4.71, p = **0.025**, η^2= 0.76*	*F (3, 11.8) = 5.99, p = **0.01**, η^2= 0.72*	*F (3, 11.48) = 2.18, p = 0.145, η^2= 0.58*
	40	*F (3, 11.53) = 4.32, p = **0.028**, η^2= 0.88*	*F (3, 11.17) = 5.91, p = **0.011**, η^2= 0.76 (WT Sham vs. WT α-MHC*)*	*F (3, 11.96) = 7.63, p = **0.0041**, η^2= 0.83 (WT Sham vs. WT α-MHC**)*	*F (3, 8.89) = 4.13, p = **0.014**, η^2= 0.77 (WT α-MHC vs. KO α-MHC*)*
	65	*F (3, 12.11) = 10.01, p = **0.0013**, η^2= 0.85 (WT Sham vs. WT α-MHC*; KO Sham vs. KO α-MHC*)*	*F (3, 10.53) = 11.44, p = **0.0012**, η^2= 0.82 (WT Sham vs. WT α-MHC**; KO Sham vs. KO α-MHC*)*	*F (3, 11.95) = 17.99, p < **0.0001**, η^2= 0.94 (WT Sham vs. WT α-MHC***)*	*F (3, 9.43) = 1.38, p = 0.31, η^2= 0.6*

LVEdD, left ventricular end-diastolic dimension; LVEsD, left ventricular end-systolic dimension; FS, fractional shortening; HR, heart rate. * - marks statistically significant differences between groups, $^{\#}$ - differences from 0 day. *,$^{\#}$ (p < 0.05), **,$^{\#\#}$ (p < 0.01), ***,$^{\#\#\#}$ (p < 0.001), by robust ANOVA followed by *lincon post hoc*.

Table S5. The list of features used for PCA.

No	Feature
1	Left ventricle end-diastolic diameter (mm) (Day 0)
2	Left ventricle end-systolic diameter (mm) (Day 0)
3	Left ventricle fractional shortening (%) (Day 0)
4	Left ventricle end-diastolic diameter (mm) (Day 21)
5	Left ventricle end-systolic diameter (mm) (Day 21)
6	Left ventricle fractional shortening (%) (Day 21)
7	Left ventricle end-diastolic diameter (mm) (Day 40)
8	Left ventricle end-systolic diameter (mm) (Day 40)
9	Left ventricle fractional shortening (%) (Day 40)
10	Left ventricle end-diastolic diameter (mm) (Day 65)
11	Left ventricle end-systolic diameter (mm) (Day 65)
12	Left ventricle fractional shortening (%) (Day 65)
13	Lung siderophages (cells/mm^2)
14	Lung weight/tibia length (mg/mm)
15	Heart weight/tibia length (mg/mm)
16	*Nppa* (relative mRNA level)
17	*Nppb* (relative mRNA level)
18	Gross score (from 0-5)
19	Infiltrates (nuclei/tissue area (%))
20	Fibrosis (% of total area)
21	*Cd44* (relative mRNA level)
22	*Col1a1* (relative mRNA level)
23	*Col3a1* (relative mRNA level)
24	*Acta2* (relative mRNA level)
25	p-JNK/JNK
26	p-ERK/ERK
27	p-p38/p38
28	p-c-Fos/c-Fos
29	p-c-Jun/c-Jun
30	FAK/GAPDH
31	p-Akt/Akt
32	YAP1/GAPDH
